# Risk Factors and Lesion Patterns in Treatment-Resistant Scabies: Impact of Sex, Age, and Comorbidities

**DOI:** 10.1007/s11686-025-01055-6

**Published:** 2025-05-27

**Authors:** Arzu Ferhatosmanoğlu, Leyla Baykal Selçuk, İbrahim Etem Arıca

**Affiliations:** Department of Dermatology and Venerology, Karadeniz Technical University School of Medicine, Karadeniz Teknik Üniversitesi, Merkez, Ortahisar/Trabzon, 61080 Turkey

**Keywords:** Pruritus, Sarcoptes scabiei, Scabies, Treatment failure

## Abstract

**Purpose:**

Scabies is a common parasitic skin condition with significant morbidity. This study aimed to identify risk factors for treatment failure and analyze lesion distribution patterns in relation to sex, age, and comorbidities in patients with treatment-resistant scabies.

**Methods:**

The study included patients with dermatologist-confirmed scabies who had received at least one treatment within the past six months without clinical improvement. Clinical, sociodemographic, and cutaneous findings were evaluated.

**Results:**

A total of 246 patients were included (130 females, 52.8%; 116 males, 47.2%). Males had significantly higher rates of excoriation (*p* < 0.001), pustules (*p* = 0.047), tunnels (*p* = 0.046), and genital involvement (*p* = 0.012). Nodules were more common in individuals under 18 (*p* = 0.003), while excoriations predominated in those over 65 (*p* < 0.001). Longer pruritus duration was observed in older adults, rural residents, and patients receiving weekend home visits (*p* = 0.017, *p* < 0.001, and *p* = 0.011, respectively), and was associated with increased lesion severity. A threefold increase in abdominal involvement and a 3.33-fold increase in pustules were seen in patients receiving three or more treatments. Higher education (university or above) was linked to a 46% reduced risk of treatment-resistant scabies.

**Conclusions:**

This study identifies key demographic and clinical risk factors associated with treatment failure in scabies and underscores the need for targeted management strategies. To the best of our knowledge, it is the first to comprehensively investigate lesion distribution and clinical patterns of resistant scabies in relation to sex, age, and comorbid conditions.

## Introduction

Scabies is an itchy ectoparasitic skin disease caused by the human-specific mite Sarcoptes scabiei var. hominis [1]. Itching at night, which increases with heat, is accompanied by the presence of itching in family members and a polymorphous rash [1]. It spreads among close contacts, and high-risk groups include children, the elderly, immunosuppressed individuals, migrants, health workers, and sexually active young adults [2, 3]. The diagnosis is made clinically and/or by detecting the mite and its products using dermoscopy, videodermoscopy, or light microscopy [1].

Scabies ranks among the 50 most common dermatologic diseases worldwide, with a growing global disease burden [4, 5]. Scabies is estimated to affect over 200 million individuals at any given time [6], with approximately 455 million new cases occurring annually [7]. It is especially prevalent in tropical and resource-poor regions. A 2024 meta-analysis found a global prevalence of 11.9% (95% CI: 9.6–14.7%) with significant variability across regions [8].

The primary first-line treatments for scabies include topical agents such as permethrin, benzyl benzoate, and sulfur, as well as oral ivermectin. Treatment failure has been associated with several factors, including the host’s immune status, choice of therapeutic agent, poor adherence to treatment protocols, risk of reinfection, and the possible emergence of drug resistance [9].

In 2021, Balestri et al. reported a reduction in the clinical efficacy of permethrin. Their findings indicated that nearly two-thirds of patients who did not respond to permethrin showed clinical improvement with an alternative topical treatment, suggesting a potential specific resistance to permethrin [10]. Proposed mechanisms of permethrin resistance in Sarcoptes scabiei include increased activity of glutathione-S-transferase, increased expression of ATP-dependent transporters (such as multidrug resistance proteins) and mutations in sodium channeles [11].

Conversely, a 2022 study assessed the in vitro scabicidal activity of permethrin on S. scabiei mites collected from patients with clinically treatment-resistant scabies. The results revealed no evidence of true resistance to permethrin. Instead, the study emphasized that poor treatment adherence may be the primary factor contributing to treatment failure and the chronicity of the disease [12].

The objective of our study was to identify potential risk factors associated with treatment-resistant scabies. Furthermore, we aimed to evaluate whether the distribution and morphological patterns of scabies lesions varied according to sex, age group, and specific clinical conditions in patients with treatment failure.

## Materials and Methods

A cross-sectional survey was conducted by the Department of Dermatological and Venereal Diseases, Faculty of Medicine between 01.02.2024 and 01.12.2024. Ethics committee approval was obtained from the Scientific Research Ethics Committee of our Faculty of Medicine dated 11/01/2024 and numbered 2023/242. Patients diagnosed with scabies within the previous six months, who persisted with pruritic rashes without significant clinical improvement despite receiving the standard treatment regimen of two topical applications one week apart, and who were diagnosed with definite scabies based on clinical history, physical examination, and dermoscopic or light microscopic findings, were classified as cases of treatment-resistant scabies and were included in the study. Clinical and sociodemographic characteristics, comorbidities and cutaneous findings of these patients were analysed.

### Statistical Analysis

Data analysis was performed using SPSS 27.0. Descriptive statistics were presented as numbers and percentages for categorical variables, and as mean, standard deviation, minimum, and maximum for metric variables. Normality was tested using the One-Sample Kolmogorov–Smirnov Test. For two-group comparisons, the Student’s t-test was used for normally distributed data, and the Mann–Whitney U-test for non-normal data. The Chi-Square Test analyzed categorical differences. Logistic Regression was employed for multivariate analysis, including variables with *p* < 0.200 in univariate analysis and known risk factors. Variable interactions were assessed via a correlation matrix. The Enter Method was applied for model inclusion, with the Hosmer–Lemeshow Test for model fit and Nagelkerke R² for explanatory power. Odds ratios (OR) with 95% confidence intervals (CI) were reported, and *p* < 0.05 was considered statistically significant.

## Results

The study included 246 participants, comprising 130 females (52.8%) and 116 males (47.2%). The age of the participants ranged from 1.5 months to 98 years, with a mean age of 28.48 ± 23.81 years. The mean size was 3.86 ± 1.56 individuals, and the mean duration of pruritus was 12.09 ± 8.33 weeks.

The sociodemographic and clinical characteristics of the patients are summarized in Table 1. Among the participants, 44.3% were under 18 years of age, 44.7% were between 18 and 65 years, and 11% were over 65 years. Nocturnal pruritus was reported by 89% of patients, pruritus aggravated by heat by 48.4%, pruritus among other family members by 61%, and reduced work or school performance by 56%.

**Table 1:** Sociodemographic and clinical characteristics of the patients

Sociodemographiccharacteristics	*n*(%)
**a-** Age	
1- 1-18 years	109(44.3)
2- 18-65 years	110(44.7)
3- Over 65 years	27(11)
**b-** Gender	
1- Female	130(52.8)
2- Male	116(47.2)
**c-** EducationStatus 1-Uneducated	25(13)
2- Primary School	43(22.3)
3- Middle School	54(28)
4- High School	48(24.8)
5- University/Master’sdegree	23(11.9)
**d-** Occupation 1- Student	69(35.8)
2- Housewife	51(26.4)
3- Retired	8(4.1)
4- Unemployed	2(1)
5- Other	63(32.6)
**e-** Place of residence	
1- City	169(68.7)
2- Village-town	77(31.3)
**f-**Receiving weekend home visits from dormitory residents	
1- Yes	39(15.9)
2- None	207(84.1)
**g-** Nocturnal pruritus	
1- Yes	219(89)
2- None	27(11)
**h-** Increased itching with heat:	
1- Yes	119(48.4)
2- None	127(51.6)
**i-** Pruritus among other family members	
1- Yes	150(61)
2- None	96(39)
**j-** Decreased success at work or school	
1- Yes	70(56)
2- None	55(44)

Table 2 presents a summary of the areas affected by scabies, types of lesions observed, treatments administered, and gender-based comparisons. No significant differences were found between males and females with respect to the duration of pruritus (*p* = 0.487), presence of nocturnal pruritus (*p* = 0.182), presence of other affected family members (*p* = 0.264), decreased success at work or school (*p* = 0.306), history of previous scabies treatments (*p* = 0.272, *p* = 0.119, and *p* = 0.101, respectively), treatment limited to the affected family member (*p* = 0.555), or presence of comorbid conditions.

Pruritus exacerbated by heat was significantly more prevalent in men than in women (*p* = 0.005). Genital involvement was also more frequently observed in men (*p* = 0.012), whereas involvement of the hands was significantly less common in men (*p* < 0.001). Additionally, men exhibited higher rates of tunnels (*p* = 0.046), excoriations (*p* < 0.001), and pustules (*p* = 0.047). In contrast, the formation of papules was significantly less frequent in men (*p* < 0.001).

**Fig. 1:**
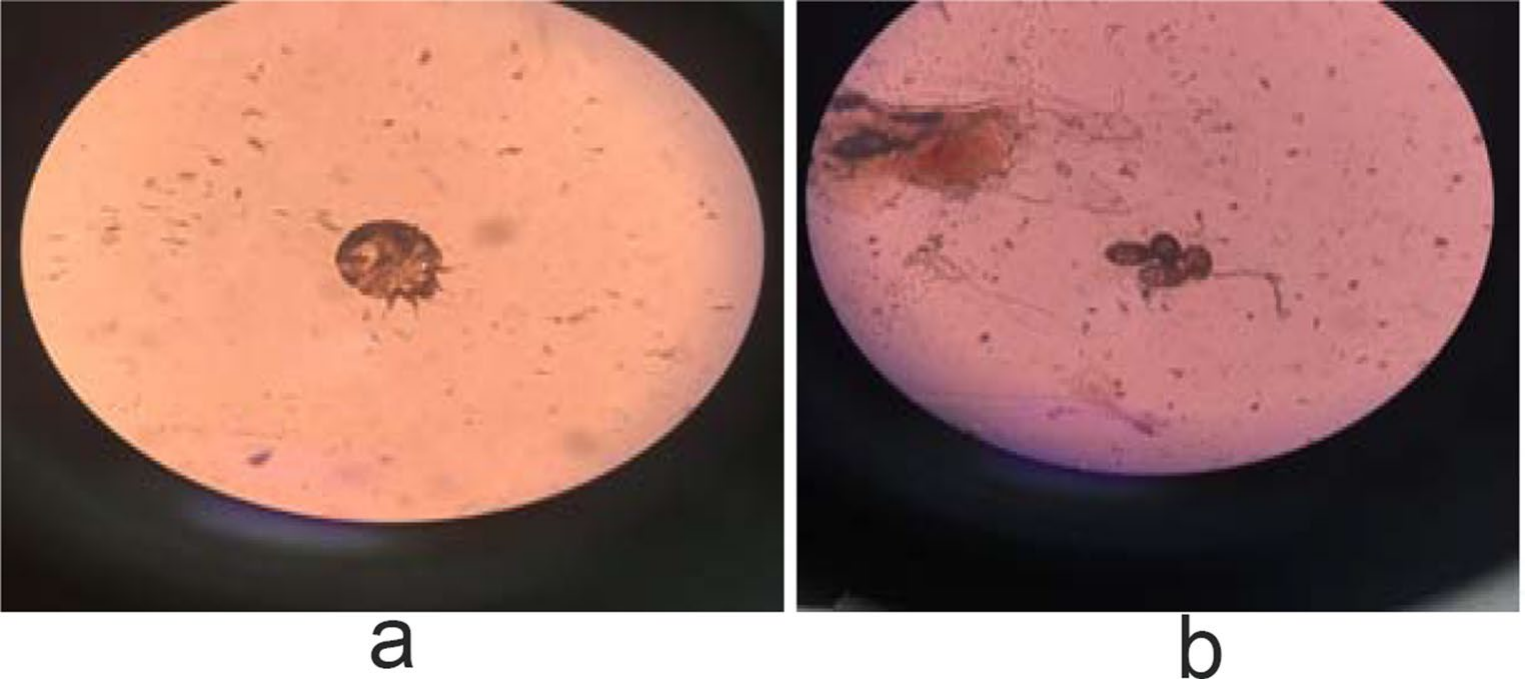
**a**. The adult stage (1**a**) and eggs (1**b**) of the parasite Sarcoptes scabiei

**Fig. 2:**
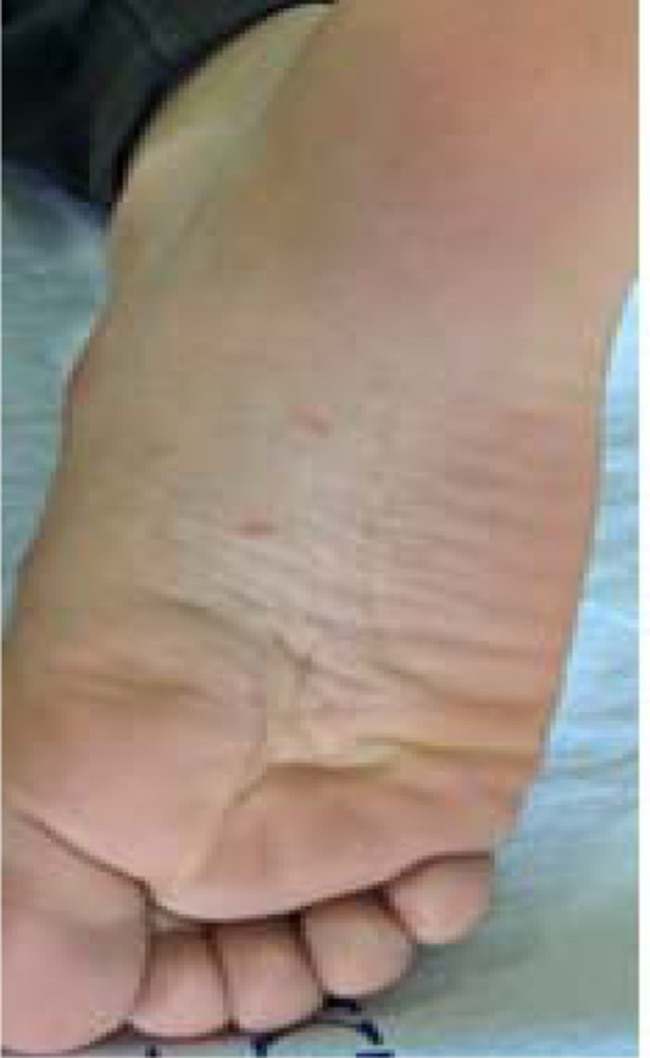
Tunnels in the plantar region

**Fig. 3:**
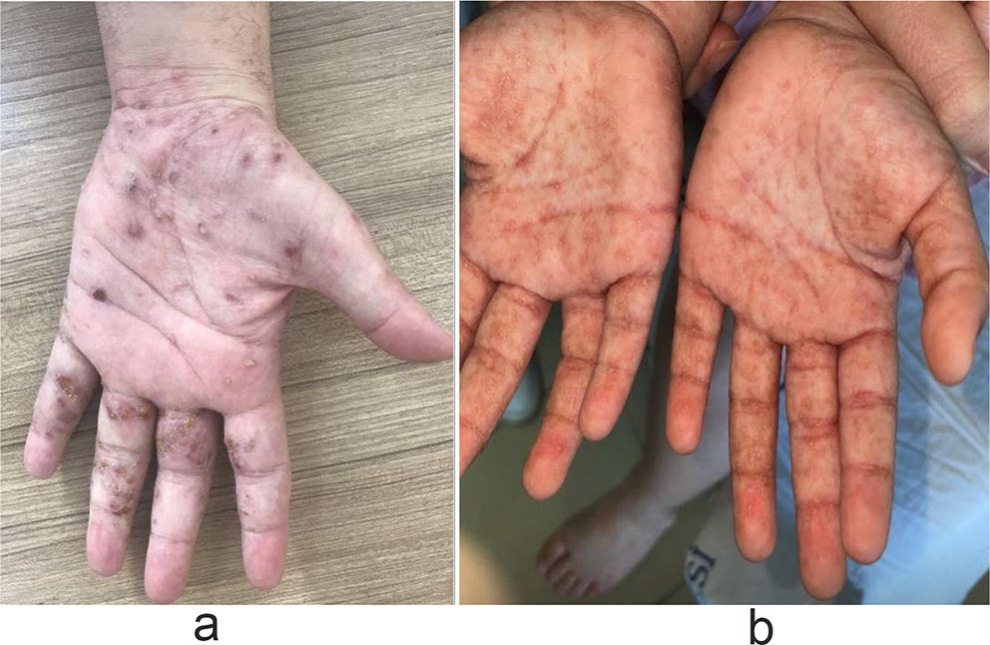
**a**: Scabetic tunnels with eczematous changes, including excoriated papules, crusting, and inflammation on the palm and interdigital spaces.; **b** Multipl tunnels on palmar regions

**Fig. 4:**
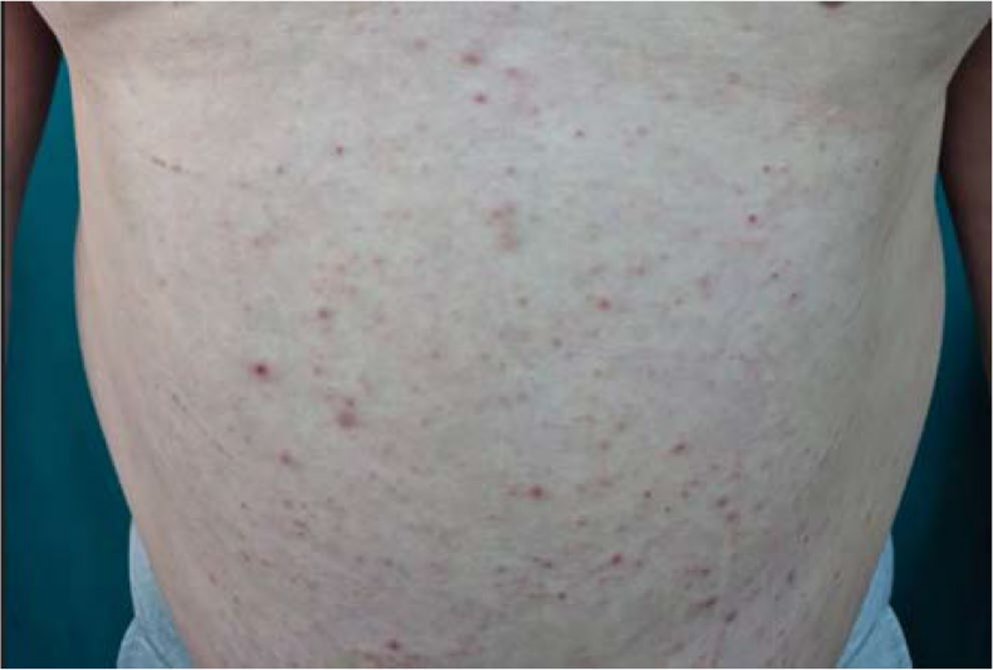
Widespread excoriations on the anterior trunk

**Fig. 5:**
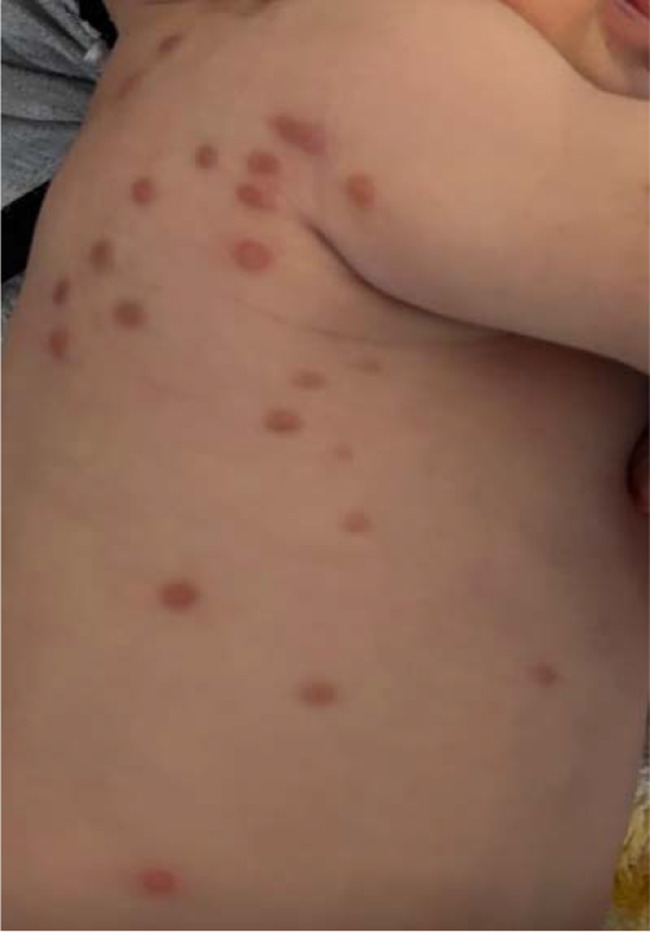
Multiple nodules on the back and lateral trunk

**Fig. 6:**
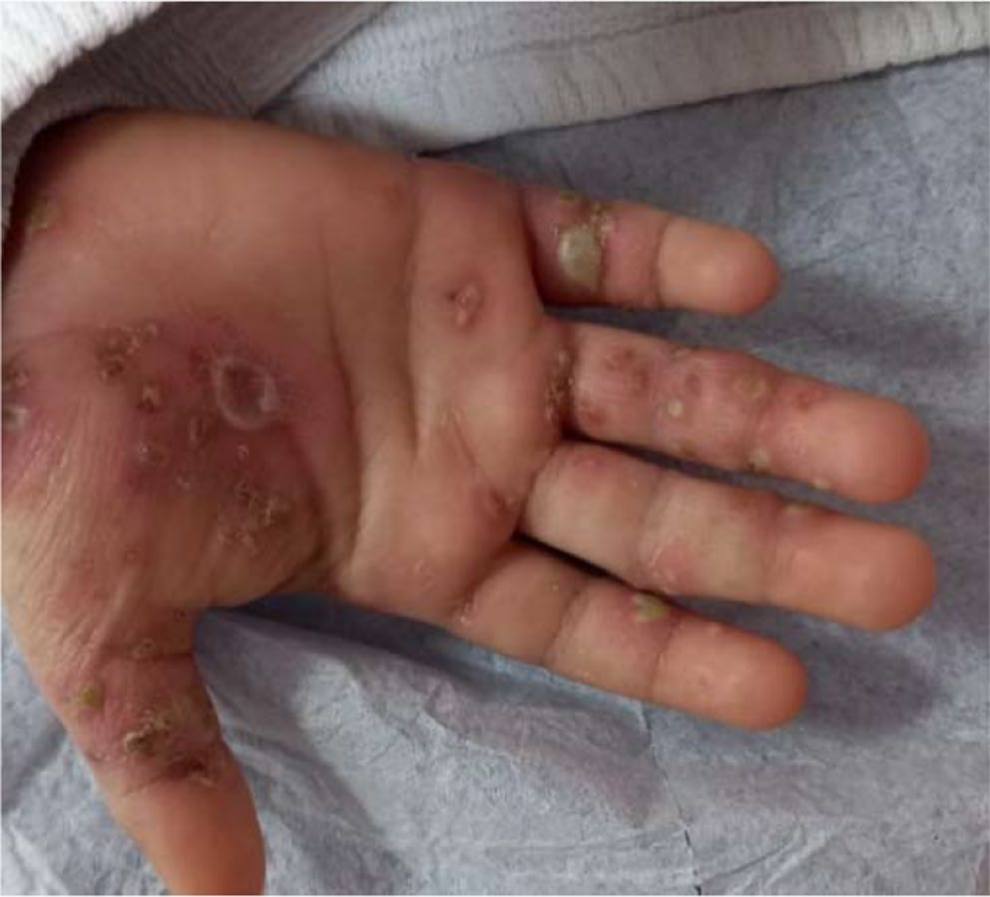
Palmar pustules secondary to bacterial superinfection

Figures 1a, b, 2, 3a, b, 4, 5 and 6 present dermoscopic and light microscopy images of the scabies parasite, along with representative cutaneous manifestations observed in affected patients.

**Table 2:** Summarisation of scabies involvement areas, lesion type and treatments received

Variables	Female *n*(%)	Male *n*(%)	Total *n*(%)	*p* value
**a-** Itching duration, (week) mean± (stdev)	12.2 ± 19.35	11.96 ± 7.04	12.09 ± 8.33	0.487
**b-** Nocturnal pruritus:				0.182
1- Yes	119(91.5)	100(86.2)	219(89)	
2- No	11(8.5)	16(13.8)	27(11)	
**c-** Pruritus exacerbated by heat				**0.005**
1- Yes	52(40)	67(57.8)	119(48.4)	
2- No	78(60)	49(42.2)	127(51.6)	
**d-**Presence of pruritus among other family members				0.264
1- Yes	75(57.7)	75(64.7)	150(61)	
2- No	55(42.3)	41(35.3)	96(31)	
**e-** Reduced performance at work or school				0.306
1- Yes	37(60.7)	33(51.6)	70(56)	
2- No	24(39.3)	31(48.4)	55(44)	
**f-** Previous treatments for scabies				
1-Permethrin				0.272
a- Yes	115(88.5)	97(83.6)	212(86.2)	
b-No	15(11.5)	19(16.4)	34(13.8)	
2- Benzyl benzoate				0.119
a- Yes	33(25.4)	40(34.5)	73(29.7)	
b-No	97(74.6)	76(65.5)	173(70.3)	
3- Topical sulfur				0.101
a- Yes	45(34.6)	29(25)	74(30.1)	
b-No	85(65.4)	87(75)	172(69.9)	
**g-** How to apply the treatment of scabies				0.555
1- Only himself	49(37.7)	48(41.4)	97(39.4)	
2- The whole family	81(62.3)	68(58.6)	149(60.6)	
**h-** Areas of skin involvement				
1- Hands	119(91.5)	83(71.6)	201(82.1)	**< 0.001**
2- Abdomen	97(74.6)	96(82.8)	193(78.5)	0.121
3- Genital	52(40)	65(56)	117(47.6)	**0.012**
4- Fold area	45(34.6)	35(30.2)	80(32.5)	0.458
5- Back	13(10)	18(15.5)	31(12.6)	0.193
6- Upper extremities	15(11.5)	15(12.9)	30(12.2)	0.739
7- Lower extremities	13(10)	14(15.5)	27(11)	0.604
**i-** Pattern of skin involvement, yes:				
1- Tunnels	95(73.1)	97(83.6)	192(78)	**0.046**
2- Excoriations	51(39.2)	73(62.9)	124(50.4)	**< 0.001**
3- Nodules	54(41.5)	41(35.3)	95(38.6)	0.319
4- Papules	59(45.4)	25(21.6)	84(34.1)	**< 0.001**
5- Eczema	29(22.3)	33(28.4)	62(25.2)	0.268
6- Pustules	14(10.8)	23(19.8)	37(15)	**0.047**
7- Vesicles	9(6.9)	9(7.8)	18(7.3)	0.802
**j-** Concomitant diseases, yes				
1- Diabetes mellitus	12(9.2)	4(3.4)	16(6.5)	0.066
2- Alzheimer’s disease	6(4.6)	7(6)	13(5.3)	0.619
3- Having a known skin disease (atopic dermatitis etc.)	6(4.6)	1(0.9)	7(2.8)	0.124
4- Being bedridden	0	1(0.9)	1(0.4)	0.472

*p value cannot be calculated

Table 3 presents the comparison of skin involvement areas and patterns according to age groups. Hands involvement was significantly more common in patients under 18 years of age (*p* < 0.001), while upper extremity involvement was more frequently observed in those aged 18 to 65 years (*p* = 0.001). In patients over 65 years of age, involvement of the abdomen (*p* = 0.008), back (*p* < 0.001), and lower extremities (*p* < 0.001) was significantly higher compared to other age groups. Additionally, nodules were significantly more frequent in patients under 18 years (*p* = 0.003), whereas excoriations were observed at a significantly higher rate in those over 65 years (*p* < 0.001).

When age groups were compared, patients over 65 years of age had the lowest proportion of family members with pruritus, which was statistically significantly lower than in the other age groups (*p* = 0.005). No significant difference was observed in the prevalence of nocturnal pruritus across age groups (*p* = 0.328). The proportion of individuals reporting pruritus exacerbated by heat was lowest in the group under 18 years of age, with a statistically significant difference compared to the other groups (*p* < 0.001).

**Table 3:** Comparison of skin involvement areas and skin involvement pattern according to age ranges

Variables	< 18 years	18–65 years	> 65 years	*p* value
Areas of skin involvement, yes				
1- Abdomen	79(72.5)	87(79.1)	27(100)	**0.008**
2- Hands	107(98.2)	70(63.6)	25(92.6)	**< 0.001**
3- Back	8(7.3)	11(10)	12(44.4)	**< 0.001**
4- Lower extremities	10(9.2)	5(4.5)	12(44.4)	**< 0.001**
5- Genital	54(49.5)	53(48.2)	10(37)	0.500
6- Upper extremities	9(8.3)	12(10.9)	9(33.3)	**0.001**
7- Fold area	42(38.5)	29(26.4)	9(33.3)	0.157
Pattern of skin involvement, yes:				
1- Excoriations	33(30.3)	68(61.8)	23(85.2)	**< 0.001**
2- Tunnels	80(73.4)	91(82.7)	21(77.8)	0.248
3- Papules	40(36.7)	40(30)	11(40.7)	0.432
4- Eczema	31(28.4)	21(19.1)	10(37)	0.091
5- Nodules	55(50.5)	31(28.2)	9(33.3)	**0.003**
6- Pustules	18(16.5)	15(13.6)	4(14.8)	0.837
7- Vesicles	12(11)	6(5.5)	0(0)	0.087
Duration of itching, weeks, (median(min.-max.))	8(4–26)	12(4–28)	12(8–60)	**0.017**
Decreased success at work or school, yes	27(56.3)	43(55.8)	*	0.965

* Over 65 years of age were not evaluated

Patients with a high school education or higher were significantly more likely to report that their entire family received scabies treatment compared to those with lower educational levels (*p* = 0.012). In contrast, place of residence (rural vs. urban) showed no significant association with family-wide treatment (*p* = 0.220).

Pruritus duration was significantly longer in individuals over 65 years of age, those living in rural areas, and those exposed to weekend visits from dormitory residents (*p* = 0.017, *p* < 0.001, and *p* = 0.011, respectively). No significant differences in pruritus duration were observed by gender or educational status (*p* = 0.487 and *p* = 0.089, respectively). Table 4 presents the differences in the types and areas of skin involvement among scabies patients, based on their symptoms and specific conditions.

**Table 4:** The differences in the types and areas of skin involvement in scabies patients depending on symptoms and specific conditions

Variables	Pruritus exacerbated by heat	Pruritus Lasting more than 3 months	Nocturnal Pruritus	Cortisone-treated patients	Diabetesmellitus	Alzh. Disease	Atopic patients
	(*p* value)	(*p* value)	(*p* alue)	(*p* value)	(*p* value)	(*p* value)	(*p* value)
**Skin involvement type**							
1**-**Excoriations	< 0.001	0.030	*	*	0.042	*	*
2-Nodules	0.001	< 0.001	*	0.045	*	0.004	*
3-Papules	*	0.016	0.025	*	*	*	*
4-Pustules	< 0.001	0.001	*	0.024	*	*	0.001
5-Tunnels	< 0.001	*	*	*	*	*	*
6-Eczema	*	0.015	0.006	*	*	0.022	< 0.001
7-Vesicules	< 0.001	*	*	0.014	*	*	< 0.001
**Areas of skin involvement**							
1- Abdomen	< 0.001	0.027	*	*	0.027	*	*
2- Hands	*	0.030	0.035	*	*	*	*
3- Genital	*	0.002	*	< 0.001	*	*	*
4- Back	0.007	0.006	*	< 0.001	< 0.001	< 0.001	*
5-Lower extremities	< 0.001	0.012	*	< 0.001	0.001	0.008	0.001
6-Upper extremities	*	*	*	0.018	0.001	0.002	*
7- Fold area	*	< 0.001	0.012	0.003	*	0.001	*

*p > 0.05

In brief, patients with diabetes mellitus and Alzheimer’s disease exhibited more extensive skin involvement, particularly on the back, lower extremities, and upper extremities. Atopic patients showed significantly higher rates of pustules, eczema, and vesicles, primarily affecting the lower extremities. Pruritus exacerbated by heat was associated with a broader distribution of lesions, including excoriations, nodules, and tunnels, with notable involvement of the abdomen and lower extremities. Patients with chronic pruritus lasting over three months displayed more widespread lesions and involvement of multiple anatomical areas. Nocturnal pruritus and prior corticosteroid use were also linked to distinct patterns of lesion distribution and types.

A total of 113 patients reported receiving two or fewer treatment cycles, while 133 patients reported undergoing three or more cycles. Among those who received two or fewer treatment cycles, 97 patients were treated with permethrin, 28 with benzyl benzoate, and 39 with topical sulfur. In contrast, 115 patients in the three or more treatment cycle group were treated with permethrin, 45 with benzyl benzoate, and 35 with topical sulfur.

We observed that the proportion of patients treated with permethrin and benzyl benzoate was significantly higher among those who received three or more treatment cycles (*p* < 0.001), whereas topical sulfur was used more frequently in the two or fewer cycle group (*p* < 0.001).

In the evaluation of skin involvement patterns in patients with treatment-resistant scabies based on their prior treatments, those treated with topical sulfur exhibited significantly less involvement in the hands (*p* = 0.024), flexural areas (*p* = 0.001), lower extremities (*p* < 0.001), upper extremities (*p* = 0.011), and back (*p* = 0.017) compared to those treated with permethrin or benzyl benzoate. However, no significant differences were observed between the groups with regard to abdominal and genital involvement (*p* = 0.182 and *p* = 0.181, respectively).

Among the total cohort, 113 patients had received two or fewer treatment cycles, while 133 patients had received three or more cycles before presenting to our clinic. Among patients who received two or fewer treatment cycles, the use of topical sulfur was more common (*p* < 0.001), whereas among those who received three or more treatment cycles, the use of permethrin and benzyl benzoate was more prevalent. Additionally, patients who had received topical sulfur at least once were less likely to report family members with pruritus (*p* = 0.025), experienced a shorter duration of symptoms (*p* = 0.001), and were less likely to have nodular lesions upon examination (*p* = 0.001) compared to those treated with other therapies.

Regression analysis revealed a three-fold increase in the likelihood of abdominal involvement and a 3.33-fold increase in the risk of pustule formation among patients undergoing three or more treatment cycles, compared to those receiving two or fewer cycles. Furthermore, a higher educational level (university or above) was associated with a 46% reduction in the risk of scabies, even among patients requiring multiple treatment cycles.

## Discussion

This study presents a comprehensive analysis of the sociodemographic and clinical characteristics, as well as the patterns of skin involvement, in patients with persistent scabies despite prior treatment. The findings underscore the influence of various factors, including gender, age, comorbidities, and treatment modalities, on the occurrence and progression of the disease. Furthermore, the study details the specific areas and patterns of skin involvement observed in cases resistant to treatment.

A 2022 study conducted in Turkey reported a higher number of male patients with scabies [13], whereas research from north eastern Brazil identified higher infection rates among women and children, who also played a more significant role in disease transmission [14]. A review from the United Kingdom (1997–2005) similarly reported a higher prevalence in women, with rates of 2.81 per 1000 population in women and 2.27 per 1000 in men, yielding a female-to-male ratio of 1.11 and indicating a slight female predominance [15]. In contrast, the present study demonstrated an almost equal gender distribution and a wide age range among affected individuals, underscoring the widespread prevalence of scabies across all demographic groups.

In a study in which 1261 individuals were evaluated, the rate of individuals over 60 years of age was 12.5%. Similarly, in this study, it was observed that individuals aged 0–65 years were mostly affected, and this may be due to the fact that individuals over 65 years of age have less contact with the external environment [16].

In this study, higher educational level (university or above) was found to be associated with a 46% reduction in the risk of developing scabies, even among patients requiring more than one course of treatment. This effect could be attributed to factors such as improved socioeconomic status, residing in less crowded environments and an increased likelihood of receiving family-wide treatment, which was significantly more common among individuals with higher education levels (*p* = 0.012), as shown in our study.

Pruritus is commonly reported among patients with scabies, with prevalence rates ranging from 90 to 99% according to current literature [17]. Nair et al. reported nocturnal exacerbation of pruritus in nearly 80% of cases [18]. Additionally, a recent Korean study found that 40.2% of patients experienced increased itching in response to heat [19]. In the present study, nocturnal pruritus was observed in 89% of patients, while heat-aggravated pruritus was reported by 48.4%, findings that are consistent with existing literature.

Genital lesions associated with scabies are frequently reported in males [20]. In the present study, genital involvement was found to be significantly higher in males compared to females (*p* = 0.012). Additionally, the rates of excoriation (*p* < 0.001), pustule formation (*p* = 0.047), and tunnel lesions (*p* = 0.046) were significantly higher among male patients. This may be attributed to the greater likelihood of moderate to severe scabies in men, as well as the higher median number of lesions reported in the literature [20].

In our study, hand involvement was observed in 82.1% of individuals, consistent with previous reports [20, 21]. However, involvement of the abdomen (78.5% vs. 23.5%) and genital region (47.6% vs. 16.3%) was notably higher compared to other studies [20]. The findings from our study may be valuable for the more detailed evaluation of these areas in patients with treatment-resistant scabies.

Hands involvement was significantly more common in individuals under 18 years of age (*p* < 0.001), whereas upper extremity involvement was more frequently observed in those between 18 and 65 years (*p* = 0.001). In patients over 65 years, higher rates of involvement were noted in the abdomen (*p* = 0.008), back (*p* < 0.001), and lower extremities (*p* < 0.001) compared to other age groups. Additionally, nodules were significantly more frequent in individuals under 18 years (*p* = 0.003), while excoriation was notably higher among those over 65 years (*p* < 0.001). To our knowledge, this is the first study to classify regional involvement and cutaneous manifestations by age group in patients with treatment-resistant scabies, providing valuable information to support the clinical evaluation and management of such cases.

In this study, the duration of pruritus was significantly longer in individuals over 65 years of age, those living in rural areas, and those receiving weekend visits than dormitory residents (*p* = 0.017, *p* < 0.001 and *p* = 0.011, respectively). This may be attributed to older adults’ limited access to healthcare services and higher transmission rates in areas with lower socioeconomic status and communal living environments. Numerous studies in the literature have similarly reported increased scabies prevalence in rural areas, low-income groups, and crowded living conditions [18, 22, 23]. These findings highlight the need for targeted education and healthcare interventions aimed at these vulnerable populations.

In one study, quality of life was moderately to severely affected in 72.2% of patients [24]. Additionally, scabies among school-aged adolescents and young adults may impair academic performance [25]. The findings of this study further support the literature, highlighting the high prevalence of nocturnal itching (89%) and its debilitating impact on work or school performance (56%), emphasizing the significant effect of scabies on quality of life.

A recent study found that the quality of life was moderately to severely affected in 72.2% of patients [24]. Moreover, scabies has the potential to impair academic performance among school-aged adolescents and young adults [25]. In our study, we observed a high prevalence of nocturnal pruritus (89%) and a significant decline in work or school performance (56%), which is consistent with the existing literature and underscores the substantial impact of scabies on overall quality of life.

When comparing treatment-resistant scabies patients based on their previous therapies, those who received topical sulfur treatment showed less widespread lesion distribution and fewer nodular lesions. Considering the suspected permethrin resistance reported in the literature [10, 11], these findings may support prioritizing topical sulfur among topical treatment options; however, larger-scale studies directly comparing these agents are needed to draw more definitive conclusions.

## Conclusion

This study provides valuable insights into the sociodemographic and clinical characteristics of patients with persistent scabies, emphasizing factors such as gender, age, comorbidities, and treatment response. To the best of our knowledge, there is no other study investigating the areas of skin involvement and patterns of involvement in patients with resistant scabies, both in terms of gender, age groups, and those with special comorbidities (such as Alz., DM).

The observed threefold increase in abdominal involvement and 3.33-fold increase in pustules among patients who received three or more treatments may indicate that these clinical findings warrant greater attention in treatment-resistant cases. Additionally, higher education levels were associated with a reduced risk of developing scabies with treatment failure, likely due to improved socioeconomic status and adherence to whole-family treatment protocols.

Age-specific patterns of regional involvement and morphological findings further underscore the complexity of scabies presentation across different demographic groups. The study also confirms the significant burden of pruritus, particularly nocturnal itching, on quality of life and daily functioning. The increased prevalence of scabies in rural areas and among older individuals with limited healthcare access highlights the need for targeted interventions in these vulnerable populations.

Given the chronicity and treatment resistance observed in some cases, comprehensive management strategies—including early diagnosis, appropriate treatment, and patient education—are essential to improving outcomes and reducing transmission. Future research should focus on optimizing treatment protocols and addressing socioeconomic disparities to enhance scabies control and prevention efforts.

## Data Availability

No datasets were generated or analysed during the current study.
